# Supplementary material for: A Likelihood Approach for Real-Time Calibration of Stochastic Compartmental Epidemic Models
Source: PLoS Comput Biol. 2017 Jan 17;13(1):e1005257. doi: 10.1371/journal.pcbi.1005257 (PMC5240920; doi:10.1371/journal.pcbi.1005257)
Supplement: S1 File — (TAR.GZ) [file pcbi.1005257.s014.tar.gz › HSPH_Online-SI-Revision/EnKF07/n1-severe/n1-severe_EnKF_table-4.pdf]

1	R0	{1.51081, 13.7078}	{2.30515, 5.54217}	{{0.634118, 0.823143}, {3., 13.9575}}	{81.3913, 61.9482}	inf	45.	81.5091	{6.7278, 208.406}	147.461	0.412293	newly	25.	93.96	{7., 165.}	316.	0.420317	Reff	1.25397	0.455645	{0.10059, 0.663958}	69.5846	ar	656.	1872.02	{1653., 2466.}	185.369
2	R0	{1.73182, 11.3873}	{2.56938, 5.83487}	{{2.13706, 0.801404}, {3., 9.55896}}	{51.2021, 51.1468}	inf	37.	59.3276	{9.8396, 107.117}	96.7031	0.279578	newly	33.	93.63	{8., 240.}	228.333	0.394787	Reff	1.5621	0.609561	{0.0763894, 2.13492}	70.1213	ar	653.	1840.33	{1620., 2507.}	181.904
3	R0	{1.50449, 10.3474}	{2.4483, 6.99422}	{{1.61087, 3.3801}, {3., 11.3129}}	{66.0899, 38.1045}	inf	32.	64.4014	{6.93424, 136.339}	141.78	0.331139	newly	23.	73.28	{5., 185.}	267.304	0.429405	Reff	1.30891	0.464413	{0.0569529, 0.669718}	68.5753	ar	651.	1855.65	{1657., 2328.}	185.046
4	R0	{1.63493, 9.1955}	{2.32151, 7.09921}	{{1.28827, 2.62703}, {3., 15.2242}}	{56.8408, 48.2013}	inf	32.	153.566	{36.7617, 348.342}	385.008	0.550255	newly	23.	139.64	{29., 387.}	512.261	0.606489	Reff	1.51231	0.521472	{0.116554, 1.21333}	67.8781	ar	663.	1633.7	{1025., 2078.}	146.971
5	R0	{1.53822, 8.05553}	{2.0912, 5.0766}	{{0.279108, 1.83962}, {3., 5.96217}}	{75.2146, 48.9757}	inf	44.	100.727	{10.8215, 184.646}	161.808	0.39148	newly	31.	103.41	{12., 278.}	261.839	0.467738	Reff	1.33671	0.380596	{0.036209, 0.602163}	71.7363	ar	621.	1607.11	{823., 2022.}	162.237
6	R0	{1.69374, 17.0793}	{2.18894, 4.61983}	{{0.598257, 1.5866}, {3., 10.9788}}	{65.6584, 72.9507}	inf	45.	49.3101	{5.39581, 45.1145}	98.2132	0.454122	newly	15.	57.94	{5., 55.}	316.533	0.431429	Reff	1.54638	0.393454	{0.0987341, 0.698146}	77.2624	ar	662.	1775.99	{1626., 1876.}	168.276
7	R0	{1.42179, 11.8031}	{2.24385, 5.12653}	{{0.61244, 1.90976}, {3., 10.6469}}	{88.5335, 56.5663}	inf	53.	66.7478	{7.20866, 110.194}	119.115	0.477081	newly	34.	71.33	{7., 87.}	170.676	0.379658	Reff	1.16871	0.422592	{0.0989551, 0.688617}	68.5888	ar	517.	1821.29	{1651., 1935.}	252.28
8	R0	{1.46683, 6.95875}	{2.21036, 7.0264}	{{1.23985, 2.69455}, {3., 13.6391}}	{63.3208, 41.7391}	inf	33.	144.882	{37.012, 348.96}	343.717	0.507949	newly	39.	128.78	{24., 269.}	244.359	0.404779	Reff	1.32308	0.511617	{0.108988, 1.20407}	66.5059	ar	653.	1610.2	{1096., 2046.}	146.763
9	R0	{1.34525, 11.505}	{2.20154, 5.13331}	{{0.612113, 1.94261}, {3., 10.8461}}	{93.7216, 55.4364}	inf	25.	48.1942	{5.60413, 62.51}	145.703	0.396081	newly	24.	55.62	{4., 62.}	182.583	0.392918	Reff	1.20669	0.389555	{0.0965578, 0.667339}	72.2103	ar	532.	1768.93	{1637., 1866.}	232.506
10	R0	{1.77958, 14.372}	{2.13696, 4.8718}	{{0.60372, 1.53188}, {3., 10.5663}}	{61.6878, 66.1021}	inf	100.	51.1853	{6.78336, 47.4936}	89.6231	0.717282	newly	47.	65.82	{5., 69.}	127.489	0.492461	Reff	1.3685	0.401301	{0.100891, 0.63531}	72.3892	ar	671.	1781.47	{1635., 1948.}	165.495
11	R0	{1.60334, 19.9548}	{2.43459, 4.62585}	{{0.887538, 0.195052}, {3., 11.8875}}	{72.6595, 76.8184}	inf	56.	43.0472	{7.72181, 101.476}	80.048	0.487292	newly	27.	89.92	{6., 348.}	286.444	0.477573	Reff	1.33398	0.563336	{0.130992, 1.25045}	63.3279	ar	617.	1759.3	{1603., 2141.}	185.138
12	R0	{1.68623, 7.58585}	{2.53046, 6.45916}	{{1.82555, 2.25578}, {3., 12.3311}}	{52.0916, 41.7881}	inf	50.	199.807	{55.0711, 589.077}	312.128	0.546705	newly	56.	205.88	{36., 495.}	282.179	0.481545	Reff	1.4822	0.663663	{0.190855, 1.17356}	61.0153	ar	640.	1576.17	{1030., 2313.}	148.245
13	R0	{1.82689, 18.0022}	{2.29812, 5.09784}	{{0.603836, 1.73555}, {3., 10.3957}}	{59.4691, 71.6821}	inf	85.	47.8773	{6.72077, 63.9709}	84.4912	0.612919	newly	30.	52.58	{5., 64.}	131.6	0.349479	Reff	1.51084	0.403446	{0.0850327, 0.663698}	74.6795	ar	673.	1773.69	{1643., 1875.}	163.55
14	R0	{1.75911, 9.16883}	{2.52885, 6.72008}	{{1.73598, 1.52633}, {3., 12.8123}}	{46.5573, 48.4539}	inf	22.	184.606	{75.6131, 317.655}	742.668	0.844423	newly	24.	194.49	{66., 372.}	713.875	0.785263	Reff	1.63773	0.65292	{0.241215, 1.05159}	62.4656	ar	661.	1545.96	{1120., 2088.}	134.076
15	R0	{1.46168, 11.3859}	{2.66493, 6.46657}	{{2.25801, 0.535963}, {3., 16.6926}}	{89.6783, 58.9969}	inf	59.	35.9913	{4.27237, 50.6309}	76.3157	0.552324	newly	40.	71.23	{4., 49.}	180.725	0.537126	Reff	1.10795	0.523338	{0.134235, 0.839806}	63.015	ar	556.	1747.87	{1625., 1770.}	214.365
16	R0	{1.53577, 11.7581}	{2.52372, 7.07207}	{{1.82974, 1.48435}, {3., 13.235}}	{65.6132, 52.0397}	inf	48.	185.879	{81.839, 324.001}	293.521	0.530066	newly	31.	187.33	{55., 353.}	509.387	0.660718	Reff	1.38834	0.63036	{0.192115, 1.03}	59.2101	ar	625.	1565.77	{1139., 2092.}	150.523
17	R0	{1.70698, 9.72924}	{2.58855, 7.20063}	{{1.89697, 1.73355}, {3., 13.227}}	{53.4156, 50.5196}	inf	51.	190.001	{75.8615, 331.241}	281.388	0.522841	newly	41.	189.41	{43., 388.}	370.756	0.564531	Reff	1.46288	0.626576	{0.120541, 1.02185}	62.8515	ar	675.	1556.19	{1108., 2127.}	130.787
18	R0	{1.5973, 14.931}	{2.55787, 7.18856}	{{1.72261, 2.56448}, {3., 13.0474}}	{60.1594, 54.6312}	inf	33.	193.899	{78.705, 332.155}	494.428	0.696079	newly	22.	188.44	{44., 379.}	762.	0.812579	Reff	1.48229	0.672746	{0.239716, 1.16543}	61.2507	ar	632.	1572.22	{1199., 2023.}	148.968
19	R0	{1.87468, 17.004}	{2.65023, 5.25374}	{{1.81138, 1.46769}, {3., 6.75029}}	{44.5616, 69.1029}	inf	109.	60.3436	{3.22029, 88.3978}	103.738	0.858986	newly	48.	96.04	{3., 160.}	208.667	0.643414	Reff	1.37227	0.600688	{0.172092, 1.27764}	59.8879	ar	692.	1803.83	{1605., 2186.}	160.669
20	R0	{1.84715, 12.531}	{2.61013, 6.43503}	{{1.80584, 2.22956}, {3., 14.6758}}	{42.4666, 57.7139}	inf	87.	38.826	{5.18299, 48.3832}	89.7598	0.732085	newly	54.	42.	{6., 63.}	96.7407	0.579657	Reff	1.37797	0.521046	{0.134437, 0.824899}	65.1369	ar	661.	1726.66	{1599., 1965.}	161.219
21	R0	{1.75938, 13.5959}	{2.55641, 7.37912}	{{1.84561, 1.97675}, {3., 13.4993}}	{47.3428, 52.8332}	inf	51.	177.224	{75.4119, 321.44}	256.089	0.501361	newly	26.	165.91	{43., 315.}	542.962	0.676347	Reff	1.52538	0.613142	{0.112792, 1.03996}	63.7841	ar	620.	1565.18	{1107., 2077.}	152.539
22	R0	{1.63456, 10.7469}	{2.6944, 8.6056}	{{2.24693, 0.791661}, {3., 16.6224}}	{69.8896, 47.2537}	inf	101.	41.5907	{4.36557, 54.1839}	82.8809	0.729311	newly	65.	60.3	{2., 53.}	125.969	0.750486	Reff	1.27823	0.608903	{0.120458, 1.02978}	62.5169	ar	654.	1786.3	{1645., 1870.}	173.135
23	R0	{1.62702, 19.1392}	{2.4324, 7.00234}	{{1.58488, 4.35506}, {3., 10.128}}	{54.4866, 63.4136}	inf	73.	60.1574	{4.43402, 85.6743}	94.3828	0.559779	newly	31.	64.62	{3., 87.}	171.806	0.427141	Reff	1.28209	0.467036	{0.045312, 0.679558}	67.8742	ar	600.	1856.82	{1653., 2303.}	209.47
24	R0	{1.70466, 12.1202}	{2.63673, 5.52095}	{{1.79944, 1.66474}, {3., 9.80336}}	{56.3574, 54.4484}	inf	59.	35.7202	{4.13876, 71.6006}	90.303	0.693799	newly	37.	60.63	{3., 102.}	165.919	0.636005	Reff	1.25293	0.614942	{0.0799595, 1.26392}	61.3026	ar	613.	1800.25	{1613., 2493.}	193.679
25	R0	{1.61735, 11.3169}	{2.79001, 6.83482}	{{2.43134, 0.773364}, {3., 14.1143}}	{72.5053, 51.6512}	inf	88.	29.2426	{14.8126, 38.673}	82.2667	0.73688	newly	70.	32.83	{6., 45.}	91.2429	0.750997	Reff	1.2583	0.456984	{0.114493, 0.596312}	64.7994	ar	681.	1654.82	{1565., 1816.}	142.999
26	R0	{1.56977, 12.3035}	{2.03848, 5.09237}	{{0.263076, 1.95574}, {3., 5.88879}}	{71.3896, 62.3927}	inf	74.	99.7598	{11.201, 168.5}	79.7196	0.300812	newly	43.	100.61	{11., 187.}	168.349	0.396898	Reff	1.30605	0.385802	{0.0365385, 0.593718}	70.7	ar	594.	1635.18	{1396., 1883.}	176.215
27	R0	{1.46317, 5.8298}	{2.52552, 5.73673}	{{2.1479, 0.612676}, {3., 9.74479}}	{75.3911, 39.7431}	inf	28.	62.6306	{10.7449, 103.973}	154.597	0.360189	newly	37.	108.84	{10., 321.}	245.676	0.438279	Reff	1.28027	0.705286	{0.111742, 2.14575}	66.8423	ar	586.	1832.27	{1605., 2504.}	213.275
28	R0	{1.70002, 14.9806}	{2.56587, 6.59905}	{{1.8747, 1.06713}, {3., 13.1162}}	{53.1572, 59.1924}	inf	49.	195.619	{71.1746, 337.548}	306.846	0.524758	newly	27.	197.64	{41., 416.}	637.111	0.717357	Reff	1.54702	0.617421	{0.146362, 1.07724}	63.1656	ar	652.	1552.25	{1129., 2091.}	139.437
29	R0	{1.52729, 16.1014}	{2.63303, 6.22548}	{{1.80554, 2.63519}, {3., 11.012}}	{76.2529, 62.733}	inf	86.	41.0015	{4.97736, 45.3302}	93.8831	0.755975	newly	43.	51.46	{5., 65.}	133.86	0.567047	Reff	1.16379	0.565648	{0.110763, 1.07353}	58.693	ar	592.	1753.44	{1615., 2005.}	196.189
30	R0	{1.72795, 13.5635}	{2.68081, 6.5564}	{{2.259, 0.775317}, {3., 15.8202}}	{61.3741, 58.6612}	inf	101.	37.8253	{4.33221, 52.1605}	81.1177	0.752368	newly	52.	43.1	{4., 51.}	95.8462	0.577911	Reff	1.1992	0.538523	{0.131749, 0.796355}	65.8039	ar	668.	1769.51	{1639., 1876.}	164.897
31	R0	{1.51591, 18.1544}	{2.30954, 5.67142}	{{0.65372, 1.66712}, {3., 13.278}}	{79.8381, 68.7601}	inf	41.	83.2749	{6.26475, 185.951}	169.606	0.427445	newly	13.	82.05	{5., 192.}	553.615	0.525608	Reff	1.27185	0.417354	{0.102871, 0.662885}	71.2539	ar	541.	1828.14	{1631., 2134.}	237.919
32	R0	{1.4282, 10.3534}	{2.50513, 5.4115}	{{2.1293, 0.457053}, {3., 10.2379}}	{80.5082, 51.7575}	inf	21.	74.0274	{11.3701, 173.912}	270.567	0.423636	newly	8.	120.62	{11., 314.}	1414.5	0.807569	Reff	1.31109	0.655848	{0.0813572, 2.12717}	68.205	ar	521.	1825.59	{1582., 2500.}	250.658
33	R0	{1.56693, 11.3972}	{2.57333, 6.9657}	{{1.91336, 1.0609}, {3., 12.9616}}	{66.6953, 51.3011}	inf	51.	192.451	{41.9737, 344.433}	289.495	0.516651	newly	36.	179.37	{31., 384.}	408.306	0.562682	Reff	1.38203	0.600743	{0.137885, 1.06402}	62.3832	ar	695.	1531.76	{1069., 2095.}	122.714
34	R0	{1.50675, 19.8823}	{2.49874, 5.79355}	{{0.962119, 0.938367}, {3., 8.53684}}	{81.9999, 70.8608}	inf	72.	78.0649	{6.37, 179.186}	116.71	0.563376	newly	29.	91.91	{5., 204.}	273.897	0.446152	Reff	1.0502	0.477153	{0.1006, 0.698147}	66.7855	ar	587.	1776.42	{1594., 2017.}	202.627
35	R0	{1.72889, 7.76068}	{2.53529, 7.3231}	{{1.83669, 1.9722}, {3., 13.5007}}	{48.5316, 50.5301}	inf	36.	184.811	{73.9763, 321.031}	419.012	0.636626	newly	45.	180.52	{44., 338.}	308.356	0.489207	Reff	1.55946	0.631057	{0.209851, 1.04241}	63.0926	ar	650.	1565.84	{1109., 2139.}	140.898
36	R0	{1.62644, 4.12303}	{2.4532, 6.40507}	{{2.07188, 2.66612}, {3., 12.0568}}	{57.6633, 77.16}	inf	30.	213.247	{68.8732, 400.044}	614.216	0.759622	newly	61.	256.11	{75., 643.}	326.443	0.504082	Reff	1.31091	0.927642	{0.316773, 1.90742}	46.6292	ar	652.	1578.49	{1072., 2180.}	142.873
37	R0	{1.70878, 15.8072}	{2.79354, 7.07131}	{{2.4367, 3.60273}, {3., 14.217}}	{63.4813, 55.2653}	inf	96.	31.9228	{2.59843, 43.1992}	81.4073	0.838789	newly	49.	38.07	{1., 81.}	103.	0.785923	Reff	1.21665	0.457775	{0.206062, 0.593213}	66.6905	ar	674.	1704.79	{1590., 1923.}	152.936
38	R0	{1.58967, 6.80568}	{2.25108, 7.19377}	{{1.24187, 3.70745}, {3., 14.1381}}	{56.2105, 41.6091}	inf	44.	145.786	{26.3541, 344.502}	245.611	0.438362	newly	51.	129.25	{19., 279.}	176.686	0.357352	Reff	1.3846	0.47651	{0.0859588, 1.13857}	68.5149	ar	610.	1601.84	{990., 2143.}	163.187
39	R0	{1.344, 8.19498}	{2.25414, 5.25197}	{{0.619853, 2.43438}, {3., 10.8983}}	{94.4632, 48.736}	inf	37.	56.354	{5.78538, 107.814}	117.709	0.385247	newly	32.	62.94	{6., 67.}	158.5	0.4131	Reff	1.1545	0.402455	{0.097888, 0.667517}	67.5597	ar	551.	1811.35	{1663., 1952.}	228.739
40	R0	{1.61509, 7.90825}	{2.51931, 6.50487}	{{2.11632, 2.60502}, {3., 12.3822}}	{63.941, 38.0748}	inf	38.	212.404	{51.7591, 392.597}	464.627	0.665688	newly	48.	234.83	{69., 609.}	395.646	0.568655	Reff	1.39544	0.863181	{0.240327, 1.52418}	50.384	ar	581.	1522.3	{894., 2214.}	165.129
41	R0	{1.60017, 5.90605}	{2.05916, 4.76359}	{{0.200869, 1.8128}, {3., 5.81925}}	{70.7349, 35.3073}	inf	46.	101.874	{9.91553, 232.678}	158.643	0.397179	newly	81.	111.91	{12., 283.}	104.975	0.413702	Reff	1.38735	0.367252	{0.0214372, 0.59912}	74.78	ar	661.	1617.95	{1182., 2071.}	148.534
42	R0	{1.64436, 15.1337}	{2.01934, 5.45161}	{{0.209551, 1.69237}, {3., 7.31038}}	{67.1065, 65.2704}	inf	52.	95.4298	{10.1703, 153.197}	118.228	0.34967	newly	26.	94.31	{10., 189.}	286.962	0.491887	Reff	1.38455	0.376942	{0.0359475, 0.640673}	72.824	ar	640.	1602.84	{963., 1992.}	156.116
43	R0	{1.48917, 11.917}	{2.80309, 7.0675}	{{2.41671, 1.36994}, {3., 15.5077}}	{88.2319, 52.8479}	inf	67.	40.2613	{16.8901, 42.2789}	92.7226	0.580285	newly	42.	50.41	{8., 37.}	137.929	0.538465	Reff	1.16602	0.487421	{0.175393, 0.715726}	62.465	ar	551.	1686.13	{1559., 1923.}	206.013
44	R0	{1.65577, 7.89317}	{2.22825, 7.045}	{{1.21081, 4.245}, {3., 13.2614}}	{54.8275, 41.1719}	inf	24.	146.878	{29.2141, 313.01}	517.928	0.632515	newly	30.	128.62	{24., 317.}	340.867	0.492097	Reff	1.52331	0.519138	{0.125915, 1.22673}	69.4019	ar	576.	1626.25	{1331., 2123.}	182.63
45	R0	{1.6909, 12.3275}	{2.4415, 6.44663}	{{1.63574, 3.74355}, {3., 9.43612}}	{51.6633, 48.7659}	inf	89.	63.4814	{7.54765, 83.0924}	93.6108	0.599048	newly	56.	67.05	{6., 102.}	124.911	0.533928	Reff	1.29354	0.434947	{0.0216527, 0.635367}	69.3821	ar	675.	1842.69	{1674., 2091.}	172.991
46	R0	{1.62351, 16.0698}	{2.23664, 4.7481}	{{0.606498, 1.9571}, {3., 10.5272}}	{70.7424, 70.4533}	inf	41.	49.5867	{6.17921, 61.1352}	95.0301	0.376351	newly	19.	57.14	{5., 62.}	232.105	0.365463	Reff	1.43681	0.386105	{0.0959629, 0.659686}	76.2832	ar	591.	1799.76	{1650., 1931.}	204.528
47	R0	{1.52372, 4.38599}	{2.60977, 7.14681}	{{2.13148, 2.54091}, {3., 12.7565}}	{75.7373, 79.9063}	inf	11.	214.211	{65.8497, 378.047}	1848.39	1.1918	newly	26.	212.16	{65., 414.}	718.538	0.783359	Reff	1.44449	0.791109	{0.230919, 1.49997}	52.0625	ar	618.	1536.67	{1055., 2035.}	150.05
48	R0	{1.44306, 11.788}	{2.32269, 4.94312}	{{0.640352, 2.14746}, {3., 9.94062}}	{85.7458, 58.0835}	inf	53.	52.1989	{4.6734, 61.0693}	97.9101	0.506383	newly	34.	54.03	{4., 70.}	128.618	0.440512	Reff	1.12126	0.383723	{0.0995096, 0.560437}	68.3323	ar	534.	1789.27	{1655., 1879.}	235.069
49	R0	{1.63624, 11.3893}	{2.26594, 7.0779}	{{1.25229, 2.73648}, {3., 14.0949}}	{55.2533, 52.2506}	inf	71.	157.798	{33.9603, 346.048}	143.351	0.311868	newly	46.	141.16	{33., 332.}	222.609	0.377936	Reff	1.39244	0.517481	{0.0991395, 1.24827}	65.7425	ar	643.	1630.81	{1072., 2091.}	154.039
50	R0	{1.46113, 11.1322}	{2.53748, 5.26339}	{{2.1309, 0.336375}, {3., 9.77738}}	{76.474, 54.726}	inf	34.	63.132	{18.3308, 125.817}	105.252	0.229214	newly	19.	113.47	{17., 242.}	507.105	0.465239	Reff	1.33547	0.626177	{0.137946, 2.12877}	66.5713	ar	501.	1850.79	{1651., 2481.}	269.503
